# Augmenting the Performance of Hydrogenase for Aerobic Photocatalytic Hydrogen Evolution via Solvent Tuning

**DOI:** 10.1002/anie.202219176

**Published:** 2023-03-27

**Authors:** Michael G. Allan, Thomas Pichon, Jade A. McCune, Christine Cavazza, Alan Le Goff, Moritz F. Kühnel

**Affiliations:** ^1^ Department of Chemistry Faculty of Science and Engineering Swansea University Singleton Park Swansea SA2 8PP Wales UK; ^2^ Univ. Grenoble Alpes CEA CNRS IRIG CBM 38000 Grenoble France; ^3^ Melville Laboratory for Polymer Synthesis University of Cambridge Lensfield Road Cambridge CB2 1EW UK; ^4^ University Grenoble Alpes CNRS DCM UMR 5250 F-38000 Grenoble France; ^5^ Dept. Hydrogen Labs and Field Tests Fraunhofer Institute for Wind Energy Systems Am Haupttor, BC 4310 06237 Leuna Germany

**Keywords:** Deep Eutectic Solvents, Hydrogen, Hydrogenase, Oxygen Tolerance, Photocatalysis

## Abstract

This work showcases the performance of [NiFeSe] hydrogenase from *Desulfomicrobium baculatum* for solar‐driven hydrogen generation in a variety of organic‐based deep eutectic solvents. Despite its well‐known sensitivity towards air and organic solvents, the hydrogenase shows remarkable performance under an aerobic atmosphere in these solvents when paired with a TiO_2_ photocatalyst. Tuning the water content further increases hydrogen evolution activity to a TOF of 60±3 s^−1^ and quantum yield to 2.3±0.4 % under aerobic conditions, compared to a TOF of 4 s^−1^ in a purely aqueous solvent. Contrary to common belief, this work therefore demonstrates that placing natural hydrogenases into non‐natural environments can enhance their intrinsic activity beyond their natural performance, paving the way for full water splitting using hydrogenases.

## Graphical abstract

Deep eutectic solvents (DESs) are shown to be superior solvents for solar hydrogen generation using natural hydrogenase enzymes. This “inDEStructible” system demonstrates that DES‐based engineered solvents stabilise hydrogenase towards oxygen and enable efficient H_2_ generation in air.

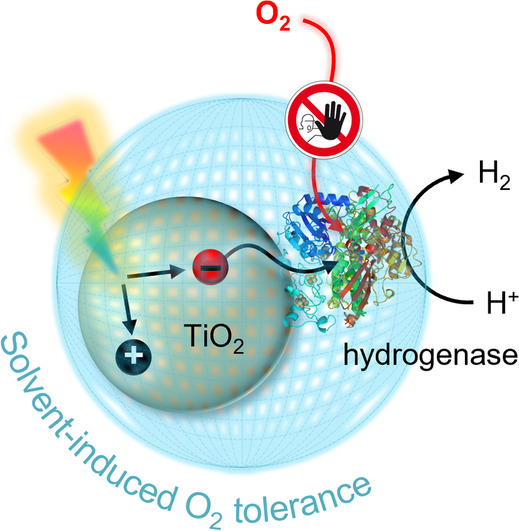

Photocatalytic water splitting is viewed as a favourable method of producing green H_2_ to combat global energy challenges without requiring large investments into electrolysers and power grids.^[1]^ Suitable systems for efficient solar H_2_ production should focus on materials which are robust, cheap, and readily available.^[2]^ A research‐intensive area in this field is the development of non‐precious co‐catalysts for the hydrogen evolution half‐reaction (HER) and its reverse hydrogen oxidation reaction (HOR).^[3]^ [NiFe], [FeFe] and [NiFeSe] hydrogenases (H_2_ases) are biological catalysts which can reversibly convert protons and electrons into H_2_ at low overpotentials without being based on precious metals.^[4]^ Solar‐driven H_2_ generation has been demonstrated using a range of H_2_ase‐photocatalyst combinations[^5^, ^6^] with [NiFeSe] H_2_ases shown to be particularly active.^[7]^ An interesting aspect of [NiFe] and [NiFeSe] H_2_ases is their O_2_ tolerance,[^7g^, ^8^] whereby they show only a partial and reversible decrease in catalytic activity under aerobic conditions. Photocatalytic H_2_ evolution in high levels of O_2_ is an important property as catalytic components in photoreactors may be exposed to O_2_ via in situ O_2_ formation resulting from water oxidation or through leakage. Despite this, O_2_‐tolerant H_2_ases show performances and lifetimes for H_2_ evolution much lower in aerobic environments versus an inert environment.^[7d]^ Solving the O_2_ sensitivity is considered a key step towards industrial application of hydrogenases.^[9]^

We recently reported on a novel approach to enabling O_2_‐tolerant H_2_ evolution through solvent design.^[10]^ Efficient photocatalytic H_2_ evolution under aerobic conditions was achieved using a Pt/carbon nitride photocatalyst employing deep eutectic solvents (DESs) with a low O_2_ solubility and diffusivity as a reaction medium. DESs have attracted attention in recent years as an alternative class of ionic liquids, as they possess low toxicities and can be prepared from cheap and readily available precursors.^[11]^ However, H_2_ases have so far not been employed outside of conventional aqueous solvents for solar H_2_ evolution due to incompatibility with different environments, particularly organic solvents.^[12]^ In the past, chemical modifications to H_2_ases have been investigated to allow them to function in organic solvents.^[13]^ Protection of H_2_ase from O_2_ has been achieved by integration with hydrogels^[14]^ and redox‐active films.^[15]^ In this work, we showcase the applicability of solvent engineering to the photocatalytic H_2_ evolution at a [NiFeSe] H_2_ase with TiO_2_ as a light‐absorber (Figure 1). We highlight for the first time not only the stability and catalytic activity of [NiFeSe] H_2_ase in organic‐based solvents for photocatalytic H_2_ production, but also a remarkable enhancement in O_2_ tolerance induced in these non‐natural solvents without any enzyme modification or catalyst redesign.

**Figure 1:**
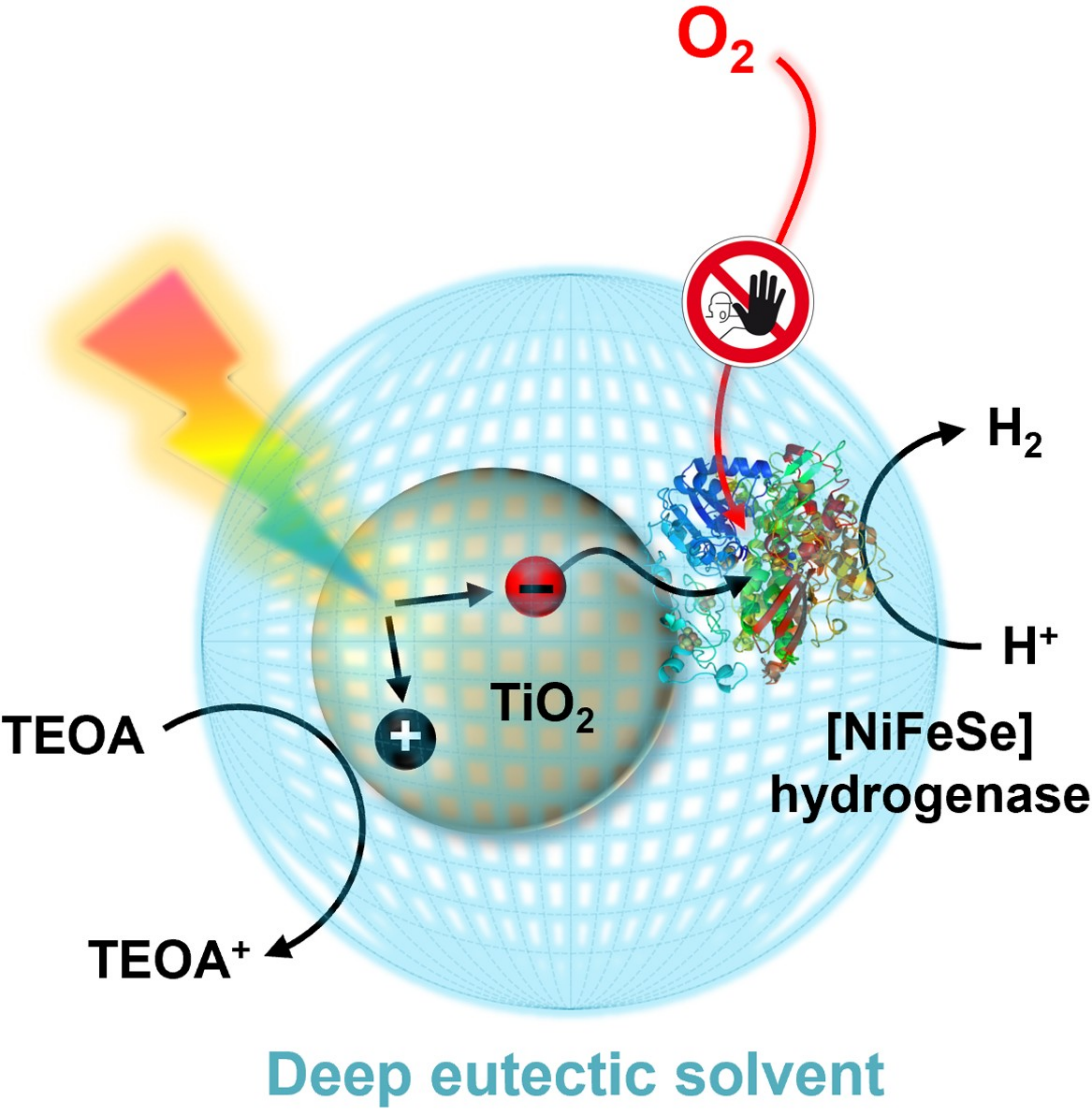
Schematic representation of the work presented here. Deep eutectic solvents induce oxygen tolerance to a TiO_2_‐hydrogenase based photocatalyst for solar‐driven hydrogen evolution under aerobic conditions.

First, we assessed the general effect of DESs on the activity of H_2_ases to investigate their potential for biophotocatalytic hydrogen evolution in non‐conventional solvents. Previous work has shown that DESs can stabilise air‐sensitive species^[16]^ and possess a high degree of biocompatibility^[17]^ with an ability to stabilise proteins.^[18]^ We chose [NiFeSe] H_2_ase from *Desulfomicrobium baculatum (Db)* as a HER catalyst for this work, due to its previously reported suitability for use as a co‐catalyst with various photocatalysts.^[7]^ The photocatalytic performance for H_2_ generation was investigated in a heterogeneous TiO_2_‐[NiFeSe] catalyst system comprised of TiO_2_ (2.5 mg mL^−1^) and *Db*[NiFeSe] H_2_ase (21 pmol) in a variety of DES‐water mixtures. Aqueous TEOA was used as an electron donor, with the pH adjusted to 7.0 prior to mixing with the DES (Table S1). Samples were irradiated with simulated solar light (AM 1.5G) at 40 °C under a continuous purge of N_2_, and H_2_ evolution was quantified by gas chromatography (see Supporting Information for full details).

In all solvents tested, we clearly observed photocatalytic H_2_ evolution, thus proving that *Db*[NiFeSe] H_2_ase retains its catalytic HER activity in DES‐based solvents. H_2_ generation by the TiO_2_‐[NiFeSe] photocatalyst system was sustained for >24 h in all solutions containing the DES glyceline (choline chloride:glycerol 1 : 2). The reaction rate increased upon increasing the water content in the solvents (Figure 2). In an 80 % vol. aq. glyceline solution, TiO_2_‐[NiFeSe] generated 24.10±0.55 μmol_H2_ (TON >1 080 000±100 000) after 24.9 h irradiation whereas the same photocatalyst in a 20 % vol. aq. glyceline solution showed a TON >4 350 000±500 000 (91.44±11.96 μmol_H2_) with an apparent quantum yield (AQE) of 2.3±0.2 % (Table S2). Comparable performance was also observed in other DESs such as ethaline (choline chloride:ethylene glycol, Figure S1). This compares favourably with an aqueous solvent, in which TiO_2_‐[NiFeSe] exhibited a TON >3 500 000±31 000 after 24.1 h under otherwise identical conditions. Remarkably, the activity in 20 % vol. aq. glyceline exceeds that observed in a purely aqueous environment by approx. 19 % under otherwise identical conditions. Negligible amounts of H_2_ were produced when TiO_2_ was irradiated in 60 %. vol. aq. glyceline without added *Db*[NiFeSe] H_2_ase (Figure S2). To the best of our knowledge, this is the first report of a natural H_2_ase functioning in an organic solvent for solar H_2_ production—and even slightly better than in purely aqueous conditions in which it evolved. It also exceeds most previous reports on photocatalytic H_2_ evolution using a [NiFeSe] H_2_ase (Table S3).

**Figure 2:**
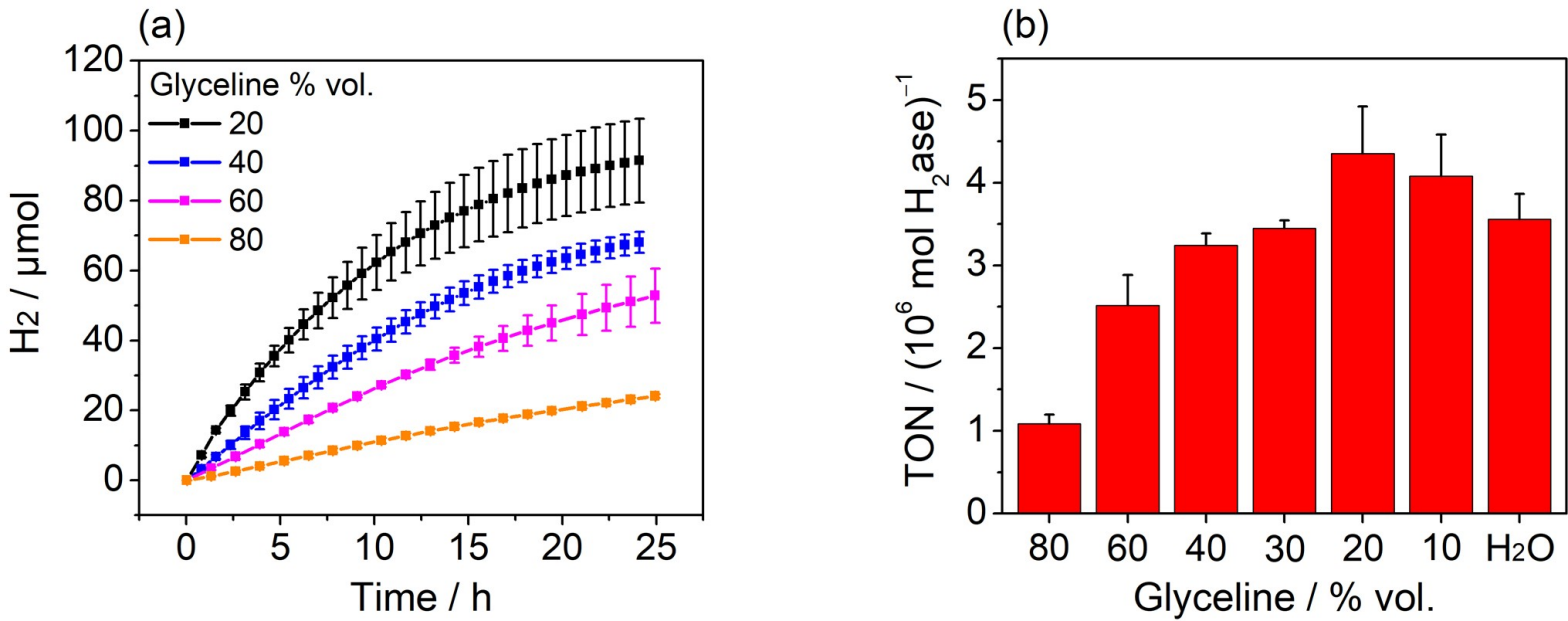
Photocatalytic H_2_ generation using a photocatalyst system based on TiO_2_ and *Db*[NiFeSe] H_2_ase in various glyceline‐water mixtures. (a) H_2_ generation over time and (b) turnover number after 24 h irradiation in solvents of varying glyceline content under inert conditions. Conditions: TiO_2_ (5.0 mg), *Db*[NiFeSe] H_2_ase (21 pmol), 2.0 mL solvent, TEOA (0.4 M), AM 1.5G, 1 sun, 40 °C, constant N_2_ purge.

Further insight into the origin of the varying H_2_ase activity depending on the DES content of the medium was sought from electrochemical measurements of *Db*[NiFeSe] H_2_ase adsorbed onto an adamantane‐modified multi‐walled carbon nanotube electrode (MWCNT)^[19]^ in varying concentrations of glyceline. Protein film electrochemistry^[20]^ shows that the enzyme retains its reversible activity towards both HER and HOR with near zero‐overpotential in the DES (Figure 3a). Upon increasing the glyceline content in the solvent, the HER current increases before gradually decreasing (Figure 3b). The presence of an HOR current arises from the production of H_2_ in the MWCNT layer upon proton reduction. Its decrease upon increasing the glyceline content might not only be caused by a decrease in HER activity but may be amplified by a decrease in H_2_ solubility with higher DES concentrations, caused by the “salting out” effect of solutions with high ionic strengths.^[21]^ A lowered H_2_ solubility in turn would mitigate the well‐documented inhibition of H_2_ase by H_2_,^[22]^ thus allowing for a higher HER activity in DESs. The initial increase in HER current is consistent with the observed increase in photocatalytic H_2_ generation reflecting the solvent effect on increasing the activity of the enzyme for proton reduction.

**Figure 3:**
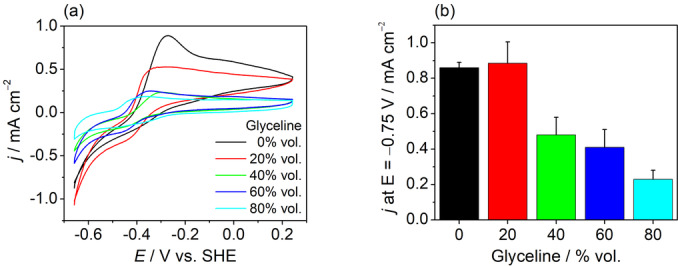
Protein film electrochemistry of *Db*[NiFeSe] H_2_ase adsorbed on a MWCNT electrode in glyceline‐water mixtures of varying composition. (a) Cyclic voltammograms and (b) observed current density at −0.75 V vs. SHE. Conditions: *Db*[NiFeSe] H_2_ase‐MWCNT working electrode, TEOA (0.4 M), pH 7.0, 25 °C, 0.01 V s^−1^ scan rate, de‐aerated solution.

To exploit the impact of the DES content in the solvents on the stability of *Db*[NiFeSe] H_2_ase under demanding conditions, the TiO_2_‐[NiFeSe] photocatalyst system was tested in the same solvents under a constant purge of air (21 % O_2_). In an aqueous aerobic sample, the photocatalyst produced 2.73±1.3 μmol_H2_ after 24.1 h irradiation (TON=130 000±60 000) corresponding to only 3.7±1.7 % of the amount produced under inert but otherwise identical conditions (Figure 4), owing to the known O_2_ inhibition of H_2_ase. In the DES‐based solutions however, a high level of oxygen tolerance is exhibited without making changes to the photocatalyst or enzyme, particularly at higher percentages of DES. In an 80 % vol. aq. glyceline solution, the O_2_ tolerance was near 90 % within experimental error with a total H_2_ production of 21.34±3.03 μmol_H2_ (TON=1 020 000±144 000) after >24 h. Similarly, the AQE of H_2_ evolution in an aerobic 60 % vol. aq. glyceline solution was determined to be 2.3±0.4 % after 1 h, identical to the value determined under anaerobic conditions. After 5.2 h irradiation in air, the AQE was still at 1.4±0.3 %, more than 70 % of the activity in inert conditions (Table S2). While higher water contents in the solvent increased the photocatalytic activity under inert conditions (vide supra), the associated decrease in DES content lowers the O_2_ tolerance. This decrease in O_2_ tolerance is also observed when other DESs are used as the reaction medium (Figure S3). The optimum compromise is observed at 60 % vol. aq. glyceline whereby TiO_2_‐[NiFeSe] continuously generates H_2_ during 72 h irradiation at a remarkable overall TOF of 60±3 s^−1^ in air, and a total TON of >1 800 000±180 000 after the 72‐h period (Figure S4). This retention of photocatalytic activity represents a major improvement over previous work showing that even O_2_‐tolerant H_2_ases in water undergo considerable inactivation during photocatalytic H_2_ evolution in the presence of air (Table S4). A [NiFe] H_2_ase from *D. vulgaris* shows 65 % retention of activity in air vs. N_2_ when embedded in a nanoporous glass plate.^[23]^ In addition, an engineered [NiFe] H_2_ase from *E. Coli* irradiated with a carbon nitride‐TiO_2_ photocatalyst system retains 20 % of its activity in air.^[24]^ [NiFe] H_2_ase from *T. Roseopersicina* covalently bound to a Ru photosensitiser was reported to maintain 11 % of its initial rate in the presence of air.^[5e]^ The *Db*[NiFeSe] H_2_ase used here has also been shown to exhibit photocatalytic performance in air when used with dye‐sensitised TiO_2_ as the photoabsorber in water, with a lowered H_2_ production rate even after just 30 minutes of exposure to air.^[7e]^

**Figure 4:**
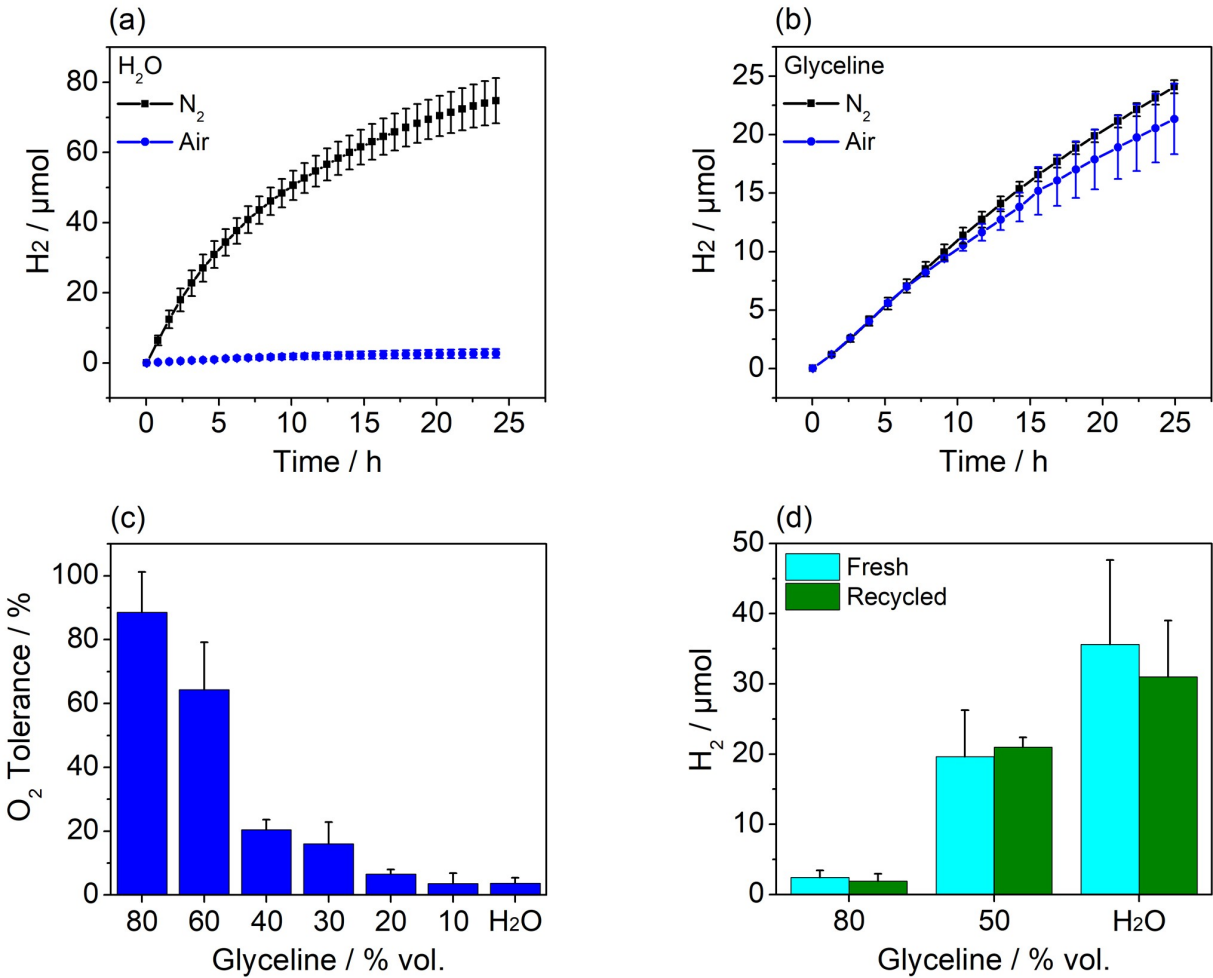
Oxygen tolerance of the photocatalytic H_2_ generation using a photocatalyst system based on TiO_2_ and *Db*[NiFeSe] H_2_ase. (a) Photocatalytic H_2_ generation in water and (b) in 80 % vol. aq. glyceline under inert conditions and atmospheric levels of O_2_. (c) Oxygen tolerance of TiO_2_‐[NiFeSe] determined from total H_2_ produced in inert and aerobic conditions after >24 h irradiation in solvents of varying concentrations of glyceline in water. (d) H_2_ produced by TiO_2_‐[NiFeSe] in fresh and resuspended solutions containing varying concentrations of glyceline after 9.3 h irradiation under N_2_. Conditions: TiO_2_ (5.0 mg), *Db*[NiFeSe] H_2_ase (21 pmol), 2.0 mL solvent, TEOA (0.4 M), AM 1.5G, 1 sun, 40 °C, constant N_2_ or air purge.

To rationalise the dependence of O_2_ tolerance on the solvent composition, we determined the O_2_ solubilities *c*(O_2_) and diffusion coefficients *D*(O_2_) in glyceline and water using stepped‐potential microwire chronoamperometry (Figure S5).^[25]^ Using the Krichevsky^[26]^ and Wilke‐Chang^[27]^ equations, we estimated c(O_2_)×D(O_2_) for the different DES/water mixtures. Table S5 shows that as the water content increases from 0 to 100 %, *D*(O_2_) increases approx. 800‐fold, while *c*(O_2_) decreases only marginally. In line with our previously reported model for the O_2_ tolerance in which O_2_ intolerance is treated as diffusion‐controlled O_2_ reduction at a spherical photocatalyst particle,^[10a]^ we observe a good correlation between c(O_2_)×D(O_2_) and the O_2_ tolerance when c(O_2_)×D(O_2_) is small, i.e. when the local concentration of O_2_ at the photocatalyst is mass transport limited and it competes with the much faster proton diffusion (Figure S6).

In addition to TiO_2_, photocatalytic H_2_ evolution at *Db*[NiFeSe] H_2_ase was further investigated using Eosin Y (EY) as a photoabsorber in aerobic and inert conditions to test the suitability of solvent tuning on a homogeneous photocatalytic system and thus the generality of this approach. In a previous report, it has been shown that EY‐*Db*[NiFeSe] is active for H_2_ evolution under visible light irradiation in the presence of atmospheric levels of O_2_, however the photoreactor was not subject to resupply of O_2_ and the activity retained in air was only 11 % relative to an inert atmosphere.^[7d]^ We observed in glyceline‐based solvents that increasing the DES content led to an increase in O_2_ tolerance of EY‐*Db*[NiFeSe] H_2_ase (Figure S7) similar to the aforementioned heterogeneous system, however the overall activity of the photocatalyst system was low. The intrinsically low activity in DESs is in line with a previous report, where photocatalytic H_2_ production at EY with synthetic HER catalysts was lower in DESs versus water but showed increased relative activity in air.^[10a]^

To investigate the origin of decreased activity at higher glyceline concentrations, solutions containing TiO_2_ and *Db*[NiFeSe] H_2_ase were subject to centrifugation, with the supernatant subsequently decanted (see Supporting Information for details). The thus obtained TiO_2_‐[NiFeSe] pellet was resuspended in a fresh solution of the solvent in question without added H_2_ase and then irradiated, and the H_2_ production was compared to a TiO_2_‐[NiFeSe] pellet resuspended in the originally decanted supernatant. H_2_ production performance of the TiO_2_‐[NiFeSe] in the recycled supernatant was similar to the performance in a fresh solution (Figure 4d). This indicates that the solvent does not hinder the adsorption of the hydrogenase enzyme to the TiO_2_ surface, allowing for efficient charge transfer from the photocatalyst to H_2_ase.

In summary, we have shown that DESs can act as alternative reaction media to water for the photocatalytic hydrogen evolution using natural hydrogenase enzymes. By tuning the water content in the DESs, both activity and stability of the H_2_ evolution activity can be increased to match and even outcompete pure water as a solvent for H_2_ evolution. The H_2_ evolution activity of TiO_2_‐[NiFeSe] in aerobic conditions is drastically improved in DESs, with nearly 90 % activity retained in air, whereas H_2_ evolution in water is almost completely quenched in air. This work shows the first instance of *Db*[NiFeSe] H_2_ase employed in organic solvents for the H_2_ evolution reaction and thus highlights the potential of solvent engineering as a novel, highly effective approach to improve natural enzyme performance. Further studies into the influence of temperature and pH on H_2_ase activity and O_2_ tolerance as well as their correlation with H_2_ and O_2_ solubilities in the respective solvent mixtures will provide key factors for designing tailored solvents in the future that can achieve a high O_2_ tolerance without lowering the H_2_ase activity.

## Conflict of interest

The authors declare no conflict of interest.

## Supporting information

As a service to our authors and readers, this journal provides supporting information supplied by the authors. Such materials are peer reviewed and may be re‐organized for online delivery, but are not copy‐edited or typeset. Technical support issues arising from supporting information (other than missing files) should be addressed to the authors.

Supporting Information

## Data Availability

The data that support the findings of this study are available in the Supporting Information of this article. Additional raw data is freely available from the Zenodo repository at https://doi.org/10.5281/zenodo.7573371.
