# Supplementary material for: Augmenting the Performance of Hydrogenase for Aerobic Photocatalytic Hydrogen Evolution via Solvent Tuning
Source: Angew Chem Int Ed Engl. 2023 Mar 27;62(22):e202219176. doi: 10.1002/anie.202219176 (PMC10946759; doi:10.1002/anie.202219176)
Supplement: Supplementary file 1 — Supporting Information [file ANIE-62-0-s001.pdf]

Angewandte Chemie
Eine Zeitschrift der Gesellschaft Deutscher Chemiker

Supporting Information

Augmenting the Performance of Hydrogenase for Aerobic Photocatalytic Hydrogen Evolution via Solvent Tuning

*M. G. Allan, T. Pichon, J. A. McCune, C. Cavazza, A. Le Goff, M. F. Kühnel**

Chemical Reagents

All chemical reagents used for this work were used as purchased and without further purification. Choline chloride (>99%), urea (analytical grade, 99.5%), ethylene glycol (>99%), glycerol (>99%), Titanium (iv) oxide (P25, Aeroxide®) triethanolamine (>98%), Eosin Y, disodium salt (certified pure), sodium chloride (>99%), potassium ferrocyanide trihydrate (crystalline, certified pure), hydrochloric acid (37% w/w, extra pure) were all purchased from Fisher Scientific. 18.2 mΩ water was used throughout the experiments. Pt microwire (d = 50 µm, 99.99%) was purchased from Advent UK.

Synthesis of Deep Eutectic Solvents

Glyceline and ethaline were prepared in accordance with literature procedures, by stirring choline chloride with glycerol and ethylene glycol, respectively in a 1:2 molar ratio at 80 °C until a homogenous liquid had formed.[1]

Purification of [NiFeSe]-H_2ase

[NiFeSe]-H_2ase was purified from the bacterium *Desulfomicrobium baculatum* as previously described.[2]

Measurement of *Db*[NiFeSe] H_2ase H_2activity

One unit (U) of hydrogenase activity was defined as the amount of enzyme which catalyses the oxidation or evolution of 1 µmol H_2 min^{-1}.

H_2 oxidation activity

The anaerobically purified *Db*[NiFeSe] H_2ase was pre-activated as follows: 200 µL of enzyme (10.5 µg mL^{-1}, 123 nM) in Tris buffer (50 mM, pH 8.5) were mixed with methyl viologen (MV, 80 µM) in a 1 mL gas-tight cuvette and placed under H_2 pressure at 30°C for 30 minutes.

H_2 uptake activity was determined spectrophotometrically at 604 nm by following the colour change of MV from a colourless oxidised form to the dark violet reduced form. All enzyme assays were performed at 30°C under anaerobic conditions in a glove box to preserve the hydrogenase enzyme from oxygen inhibition. The reaction mixture containing Tris (50 mM, pH 8.5) and MV (1 mM) in a final volume of 1 mL was first saturated with gaseous H_2 in an assay cuvette. 1 µL of pre-activated *Db*[NiFeSe] H_2ase was injected into the assay cuvette and the absorbance at 604 nm was followed. Rates of MV reduction were calculated using an absorption coefficient of 13.6 mM^{-1} cm^{-1} (with 1 equivalent of oxidised H_2 per 2 equivalents of reduced MV).

The specific H_2 oxidation activity of the *Db*[NiFeSe] H_2ase used here was 2,115±121 U mg^{-1}, corresponding to a k_{cat} of 2997 s^{-1}.

H_2 evolution activity

The anaerobically purified *Db*[NiFeSe] H_2ase (3 µL, 300 µg mL^{-1}, 3.53 µM) was diluted in sodium acetate buffer (1 mL, 100 mM, pH 4.6) to a final enzyme concentration of 0.9 µg mL^{-1} (10.5 nM) and mixed with MV (5 mM, pre-reduced with 1 molar equivalent of sodium dithionite) in a gas-tight 10 mL vial under a N_2 atmosphere in a glove box. The vials were then incubated at 30°C and 125 rpm. The production of H_2 was analysed with a PerkinElmer Clarus 500 gas chromatograph. 50 µL of the gas phase were injected at different incubation times (3, 5, 7, 10, 15, 17, 19, 25 and 30 minutes). Activities were measured between 3 and 5 minutes of incubation.

The specific H_2 evolution activity of the *Db*[NiFeSe] H_2ase used here was 172±18 U mg^{-1}, corresponding to a k_{cat} of 245 s^{-1}.

Treatment of data

All photocatalysis measurements were performed in triplicate and are given as the unweighted mean ± standard deviation (σ). σ of a measured value was calculated using Equation (S1), where n is the number of repeated measurements, x is the value of a single measurement and $\bar{x}$ is the unweighted mean of the measurements.

$$\sigma = \sqrt{\frac{\sum(x-\bar{x})^2}{n-1}} \qquad \text{(S1)}$$

Apparent Quantum Yield (AQY): Photocatalysis samples were prepared as stated above using a glass sample vial (Chromacol 10-SV, Fisher) as the photoreactor with an irradiated area A = 2.5 cm^2. Samples were purged with N_2 or air continuously during irradiation with monochromatic light using a narrow-pass filter (Oriel, λ = 405 nm, I = 5.3 mW cm^{-2}). Hydrogen was quantified by GC using the process described below. AQY was calculated according to equation S2.

$$\text{AQY (\%)} = \frac{2\text{n} \times \text{N}_\text{A} \times \text{h} \times \text{c}}{\text{t}_\text{irr} \times \text{I} \times \text{A} \times \lambda} \qquad \text{(S2)}$$

here n is the total H_2 produced per unit time, N_A is Avogadro's Constant, h is Planck's Constant, c is the speed of light, t_{irr} is the irradiation time, I is the irradiation intensity and A is the irradiated area.

Preparation of Samples

All samples used in the experiments were prepared in the following manner unless otherwise stated. TiO_2 powder (5.0 mg) was weighed into a glass sample vial (Chromacol, 10-SV) along with 2.0 mL of solvent and a stir bar. Samples were briefly vortexed and then sonicated in a sonic bath for 20 minutes. The solutions were capped with a rubber septum and purged with N_2 gas from MFCs (Bronkhorst) with a flow rate of 20 mL min^{-1} for 10 minutes to de-aerate the solutions. The samples were then introduced into a glovebox under an inert atmosphere (Saffron, O_2 level < 5.0 ppm).

In the glovebox, the septa were removed from the sample, and an aliquot of *Db*[NiFeSe] H_2ase (10 µL, 2.1 $\times 10^{-3}$ mM) was added to each sample, giving a final loading of *Db*[NiFeSe] 21 pmol H_2ase in each sample. For samples containing Eosin Y, 1 µmol (0.5 mM) of Eosin Y was used instead of TiO_2 and 10 pmol of *Db*[NiFeSe]. Samples were then re-sealed and removed from the glovebox.

Photocatalytic H_2 Generation

Samples were mounted in a water bath maintained at 40°C and stirred at 800 RPM. Each sample was connected to an individual gas supply (N_2 or air) and an inlet of the GC stream-selector valve using hypodermic needles. The sample headspace was continuously purged with the respective gas (N_2 or air) at a constant flow rate of 4 mL min^{-1} controlled by a mass flow controller (Bronkhorst); this purge was maintained throughout the experiment. After a minimum purge of 5 minutes to saturate the solution and headspace with the gas, the samples were irradiated using a solar light simulator (Thermo Oriel 92194-1000) equipped with an AM 1.5G filter (Newport) with an intensity of 1 sun. H_2 evolution was monitored by gas chromatography (Shimadzu Nexis 2030) using an auto-sampler programmed to inject 2 mL of the selected headspace purge gas stream.

Resuspension Experiments

Samples were prepared as above with TiO_2, and [NiFeSe]-H_2ase was added as described above in the glovebox. Once the enzyme was added, samples were briefly stirred in the glovebox for 30 minutes at 600 rpm. These solutions containing enzyme and TiO_2 were decanted into centrifuge tubes in the glovebox and then sealed. The centrifuge tubes were removed from the glovebox and centrifuged on a benchtop centrifuge at 5000 rpm. Pellets of TiO_2 were recovered by removal of the supernatant in the glovebox, and the pellets were then resuspended in a fresh solution of the same nature as the decanted supernatant but without hydrogenase. For samples with recycled supernatant, the supernatant originally decanted off after initial centrifugation was added again (to account for any losses during decanting) and used to resuspend the TiO_2 pellets.

Physical Measurements

Sample Analysis by Gas Chromatography (GC). Gas chromatography was performed on a Shimadzu Nexis GC-2030 gas chromatograph equipped with a barrier-discharge ionisation detector (BID) and a molecular sieve column. The total run time of the method was 5 minutes. The GC was calibrated using calibration gas (2000 ppm H_2, BOC), diluted with N_2 at different ratios using a set of mass flow controllers (Bronkhorst) to provide known concentrations of H_2. Gas samples were programmed to auto-inject into the GC via a multiport stream selector valve directing the selected

sample purge gas stream through a 2 mL sample loop before injection. H_2 evolution rates were calculated from the measured H_2 concentration in the purge gas and the purge gas flow rate. Cumulative H_2 production was calculated from the H_2 evolution rate and time passed since the previous measurement, assuming a constant H_2 evolution rate between time points. All samples were performed in triplicate (unless otherwise stated).

[NiFeSe]-H_2ase – MWCNT Electrode Preparation

N-methyl-2-pyrrolidone (NMP) dispersions of MWCNTs were prepared by 30 min sonication of 5 mg MWCNTs dispersed in 1 mL NMP until a homogeneous black suspension was obtained. Then 20 µL of the MWCNTs solution were drop-cast on a glassy carbon electrode and NMP was removed under vacuum leaving a 5-µm-thick film on the GCE. MWCNT-modified electrodes were soaked for 1 h in DMF solution containing 10 mM of 1-pyrenebutyric acid adamantyl amide and thoroughly washed with DMF and distilled water. The functionalised electrodes were then incubated with 20 µL of the enzymatic solution (1.5 mg mL^{-1}) in 50 mM sodium phosphate buffer at pH 7.6 for 1 hour at room temperature. The electrodes were finally washed with buffer and stored in the respective buffer when not in use.

[NiFeSe]-H_2ase – MWCNT Electrochemical Setup

The electrochemical experiments were carried out in a three-electrode electrochemical cell using a Biologic VMP3 Multi potentiostat. The MWCNT bioelectrodes were used as working electrodes. Pt wire was used as counter electrode and the saturated calomel electrode (SCE) served as reference electrode. The experiments were conducted at room temperature. All current densities are normalised towards the geometrical surface of the glassy carbon electrode (0.071 cm^2). Prior to experiments, the hydrogenase-modified electrodes were activated by poising the electrode at –1 V vs. SCE for ten minutes.

Determination of O_2 solubility in glyceline/water mixtures

O_2 solubility in glyceline and water was determined by stepped-potential chronoamperometry using a Pt microwire electrode as previously described.[3] The chronoamperograms were fitted with the Shoup-Szabo equation (eqn. S3) to derive $c(O_2)$ and $D(O_2)$ using a concatenate fit of three individual data sets using the Origin programme (see Fig. S5).

$$I(t) = nFlDc \times \left(\frac{\pi \times e^{-\frac{2}{5} \times \sqrt{\pi \frac{Dt}{16r^2}}}}{4\sqrt{\pi \frac{Dt}{16r^2}}} + \frac{\pi}{ln\left[\sqrt{\left(64 \times e^{-0.5772 \times \frac{Dt}{16r^2}}\right) + e^{\frac{5}{3}}}\right]} \right) \quad \text{(S3)}$$

Where I is the current, n is the number of transferred electrons, F is Faraday's constant, l is the electrode length, D is the O_2 diffusion coefficient, c is O_2 concentration, t is the time, and r is the electrode radius.

From the solubility, the Henry's constants for O_2 in water and glyceline were calculated using eqn. S4

$$k_H(O_2) = \frac{c(O_2)}{p(O_2)} \quad \text{(S4)}$$

Where $k_H(O_2)$ is the Henry constant for O_2, $c(O_2)$ is the O_2 solubility and $p(O_2)$ is the partial pressure of O_2 in air.

Using the Krichevsky[4] equation (eqn. S5), the O_2 Henry's constants for the different solvent mixtures were estimated. Eqn. S4 was used to obtain the O_2 solubilities from the calculated Herny constants.

$$ln\, k_H(O_2)_s = x_{gly} \times ln\, k_H(O_2)_{gly} + x_{H2O} \times ln\, k_H(O_2)_{H2O} \quad \text{(S5)}$$

Where $k_H(O_2)_s$ is the Henry constant for O_2 in the respective solvent mixture, x_{gly} is the mole fraction of glyceline in the solvent mixture, $k_H(O_2)_{gly}$ is the Henry constant for O_2 in glyceline, x_{H2O} is the mole fraction of water in the solvent mixture and $k_H(O_2)_{H2O}$ is the Henry's constant for O_2 in water.

Using the Wilke-Chang equation[5] (eqn. S6), the diffusion coefficients of O_2 in the different solvent mixtures were estimated.

$$D(O_2)_s = \frac{1 - x_{O2}}{\frac{x_{gly}}{D(O_2)_{gly}} + \frac{x_{H2O}}{D(O_2)_{H2O}}} \approx \frac{1}{\frac{x_{gly}}{D(O_2)_{gly}} + \frac{x_{H2O}}{D(O_2)_{H2O}}} \quad \text{(S6)}$$

Where $D(O_2)_s$ is the O_2 diffusion coefficient in the solvent mixture, x_{O2} is the mole fraction of O_2 in the solvent (assumed negligible due to the micromolar solubility), x_{gly} is the mole fraction of glyceline in the solvent mixture, x_{H2O} is the mole fraction of water in the solvent mixture, $D(O_2)_{gly}$ is the O_2 diffusion coefficient in glyceline and $D(O_2)_{H2O}$ is the O_2 diffusion coefficient in water.

Supporting Figures

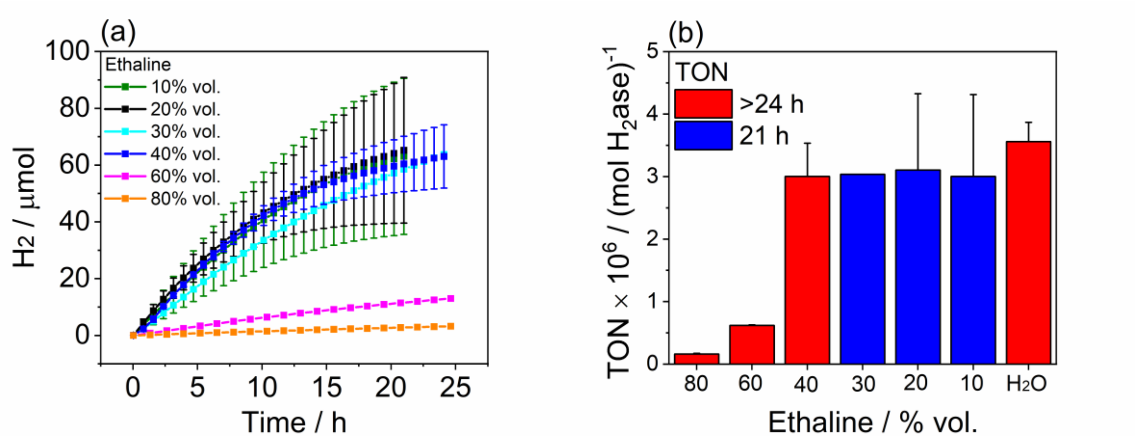

Figure S1. Photocatalytic H_2 generation using a photocatalyst system based on TiO_2 and *Db*[NiFeSe] H_2ase in various ethaline / water mixtures. (a) H_2 generation over time and (b) turnover number after more than 24 h irradiation (red) and 21 h irradiation (b) in solvents of varying ethaline content under inert conditions. Conditions: TiO_2 (5.0 mg), *Db*[NiFeSe] hydrogenase (21 pmol), 2.0 mL, TEOA (0.4 M), AM 1.5G, 1 sun, 40 °C, constant N_2 purge.

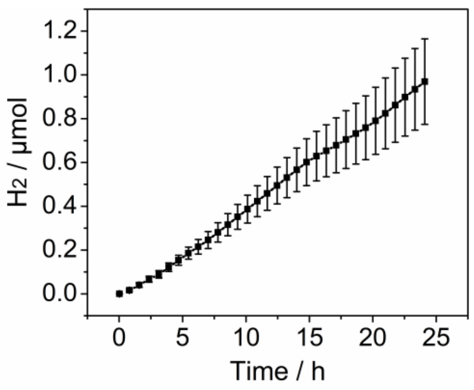

Figure S2. H_2 evolution by TiO_2 in 60% vol. glyceline in the absence of *Db*[NiFeSe] H_2ase co-catalyst. Conditions: TiO_2 (5.0 mg), TEOA (0.4 M), 2.0 mL 60% vol. aq. glyceline, 40 °C, AM 1.5G, constant N_2 purge.

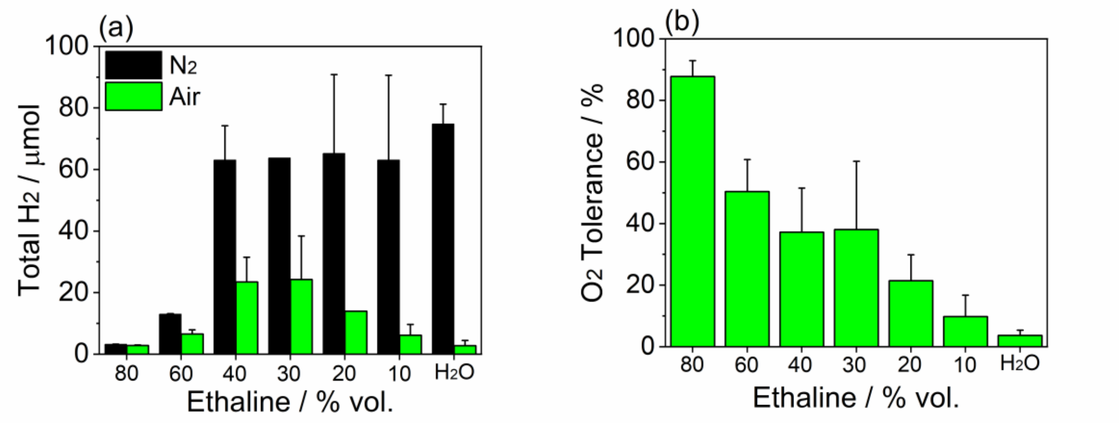

Figure S3. Photocatalytic H_2 generation using a photocatalyst system based on TiO_2 and *Db*[NiFeSe] H_2ase in solutions of varying ethaline content in water under inert and aerobic conditions. (a) Oxygen tolerance of TiO_2 and *Db*[NiFeSe] H_2ase determined from total H_2 produced in inert and aerobic conditions after > 21 h irradiation in solvents of varying concentrations of ethaline in water (b). Conditions: TiO_2 (5.0 mg), *Db*[NiFeSe] H_2ase (21 pmol), 2.0 mL solvent, TEOA (0.4 M), AM 1.5G, 1 sun, 40 °C, constant N_2 or air purge.

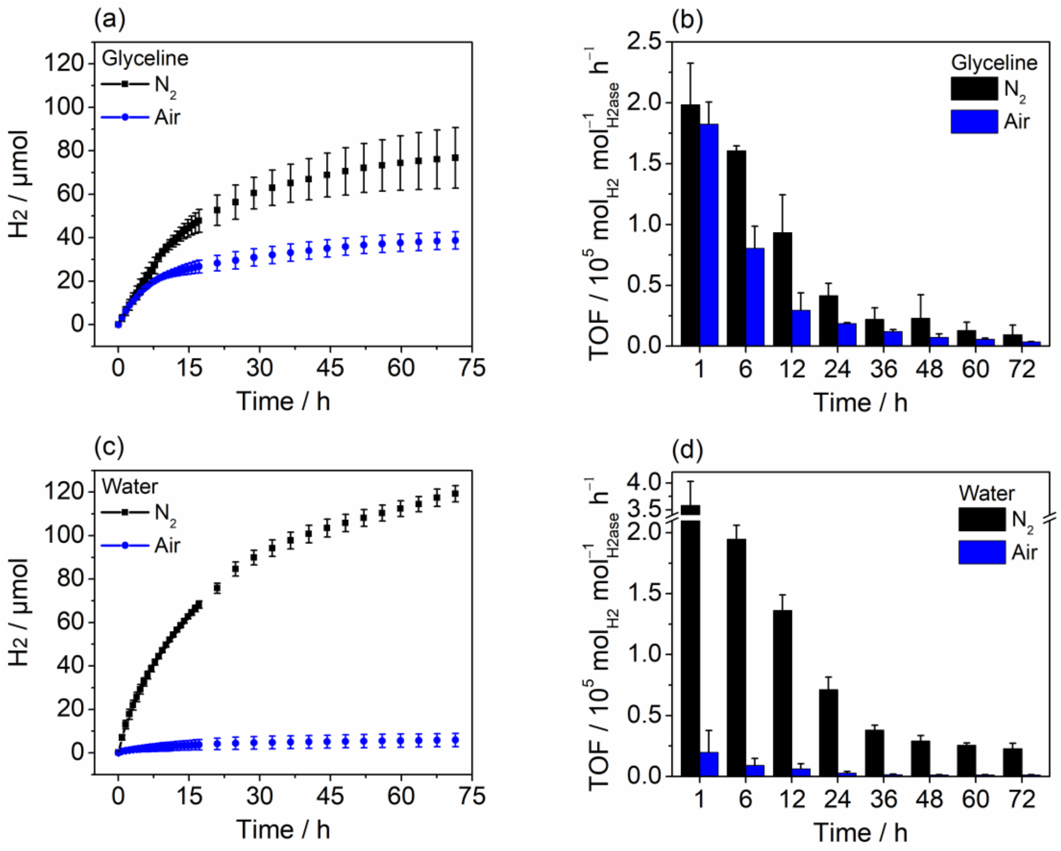

Figure S4. H_2 production by TiO_2-[NiFeSe] for over 72 hours in 60% vol. glyceline (a) and water (c) in inert and aerobic conditions. Turnover frequency of *Db*[NiFeSe] H_2ase over time in 60% vol. glyceline (b) and water (d) in inert and aerobic conditions. Conditions: TiO_2 (5.0 mg), *Db*[NiFeSe] H_2ase (21 pmol), TEOA (0.4 M), pH 7.0, 2.0 mL water or 60 %vol aq. glyceline, 40 °C, AM 1.5G, constant N_2 or air purge. For the long-term data after 16 h, points in between 4 h intervals are omitted for clarity.

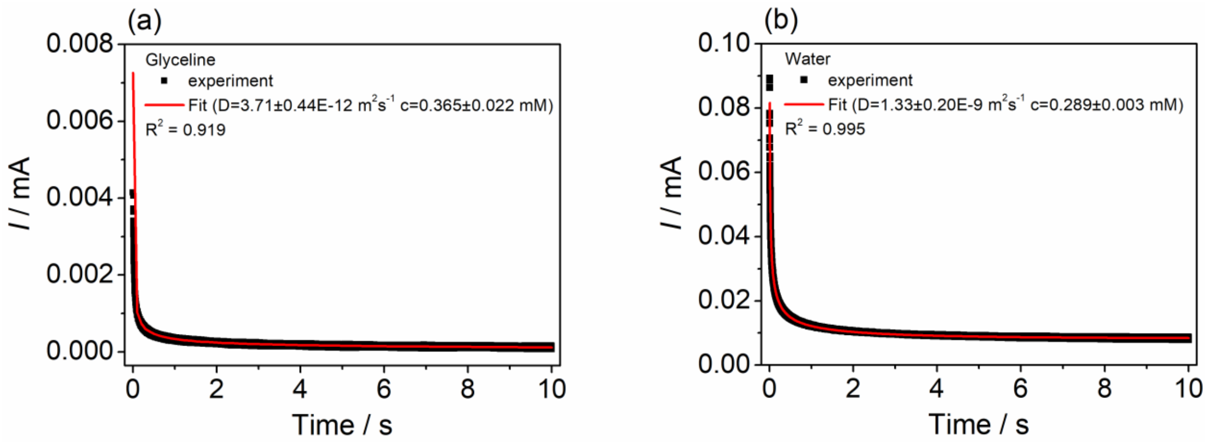

Figure S5. Determination of O_2 solubility and diffusivity in glyceline (a) and water (b) at 40°C using a Pt microwire electrode. Superposition of three independent O_2 reduction chronoamperograms (2 ms sampling rate, total run time 10 s) upon stepping from 0.2 V to −0.9 V vs. Ag/AgCl and the concatenated fit according to eqn. S3 (first two data points of each chronoamperogram were excluded from the fit).

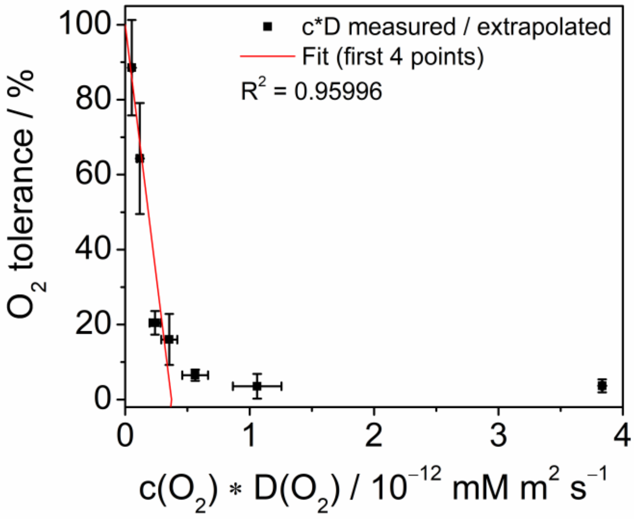

Figure S6. Correlation of observed O_2 tolerance during photocatalysis with the solubility and diffusion coefficient of O_2 in the reaction medium. Linear fitting was based on a subset with O_2 tolerance > 10%.

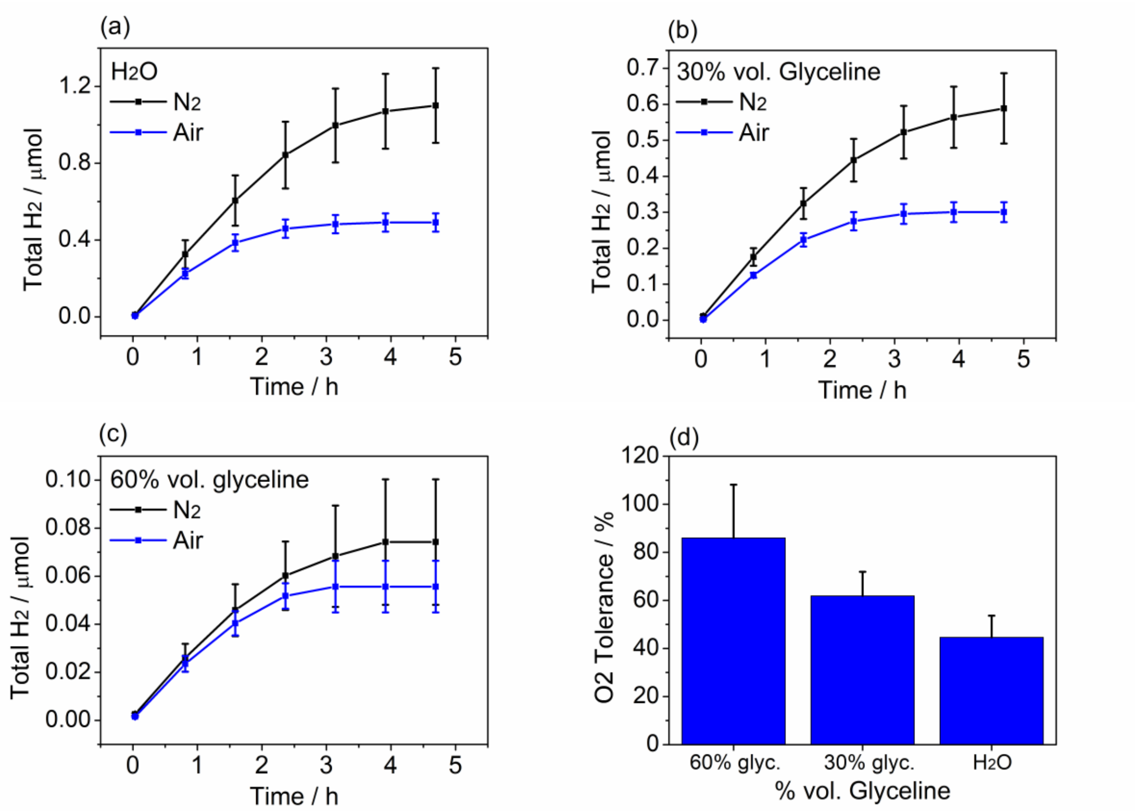

Figure S7. H_2 production by a photocatalytic system comprised of Eosin Y (EY) and *Db*[NiFeSe] H_2ase (a) in H_2O, (b) in 30% vol. aq. glyceline and (c) in 60% vol. aq. glyceline in inert and aerobic conditions. (d) O_2 tolerance of EY-*Db*[NiFeSe] in respective solvents. Conditions: EY (0.5 mM), *Db*[NiFeSe] H_2ase (10 pmol), TEOA (0.4 M), pH 7.0, 2.0 mL, 40 °C, λ > 400 nm, constant N_2 or air purge.

Supporting Tables

Table S1. Aqueous TEOA concentrations added to DES solutions. The final TEOA concentration after pH adjustment was 0.4 M.

Initial TEOA Concentration<br>M	Initial pH	Volume added<br>mL	Concentration of aq. TEOA<br>% vol.
**1.947**	7.0	0.38	20%
**1.27**	7.0	0.58	30%
**0.949**	7.0	0.78	40%
**0.755**	7.0	0.98	50%
**0.627**	7.0	1.18	60%
**0.536**	7.0	1.38	70%
**0.47**	7.0	1.58	80%
**0.416**	7.0	1.78	90%

Table S2. Apparent Quantum Yield (AQY) determination for anaerobic and aerobic photocatalytic H_2 evolution in 60% vol. glyceline. Conditions: TiO_2 (5.0 mg), *Db*[NiFeSe] H_2ase (21 pmol), TEOA (0.4 M), 2.0 mL glyceline (60% vol. in water), 40 °C, constant N_2 or air purge. A = 2.5 cm^2, λ = 405 nm, I = 5.3 mW cm^{-2}.

Time<br>h	n(H_2) – N_2<br>µmol	QE – N_2<br>%	n(H_2) – Air<br>µmol	QE - Air<br>%
1.1	1.7±0.1	2.3±0.2	1.7±0.3	2.3±0.4
2.1	3.6±0.2	2.2±0.3	3.3±0.5	1.9±0.2
3.1	5.4±0.3	2.1±0.3	4.5±0.6	1.7±0.4
4.2	7.1±0.5	2.2±0.4	6.2±0.8	1.5±0.4
5.2	8.9±0.6	2.0±0.4	7.3±0.8	1.4±0.3
10.1	15.5±1.3	1.5±0.2	11.0±1.3	0.7±0.3
15.3	21.3±1.9	1.3±0.2	13.2±2.1	0.4±0.3
23.9	29.1±2.9	1.0±0.1	15.5±3.8	0.4±0.3

Table S3. Literature examples of photocatalytic H_2 evolution using [NiFeSe] hydrogenase under inert conditions.

Photocatalyst [NiFeSe] source	Light source	TON $mol_{H2}\ mol^{-1}{}_{H2ase}$	TOF_{max} $mol_{H2}\ mol^{-1}{}_{H2ase}\ s^{-1}$	Ref
RuP-TiO_2 *D. baculatum*	Tungsten halogen lamp λ>420 nm, 45 mW cm^{-2}	190,000 (4 h)	6.6	[6]
RuP-TiO_2 *D. baculatum*	Tungsten halogen lamp λ>420 nm, 45 mW cm^{-2}	397,500 (4 h)	72±5 (45°C)	[7]
EY *D. baculatum*	AM 1.5G λ>420 nm, 100 mW cm^{-2}	50,000±3,000 (24 h)	13.9±0.7	[8]
$Ru(bpy)_3^{2+}$ *D. baculatum*	AM 1.5G λ>420 nm, 100 mW cm^{-2}	98,000±800 (24 h)	27±2	[8]
CN_x-TiO_2 *D. baculatum*	AM 1.5G 100 mW cm^{-2}	580,000±60,000 (72 h)	2.8±0.6	[9]
CD-$NHMe_2^+$ *D. baculatum*	AM 1.5G 100 mW cm^{-2}	52,000±8,000 (48 h)	1.08±0.25 ×10^{-3}	[10]
In_2S_3 *D. vulgaris*	Xe lamp 37 mW cm^{-2}	n/a	952	[11]
TiO_2 *D. baculatum*	AM 1.5G 100 mW cm^{-2}	4,350,000±500,000 (24.1 h)	122.4±10.1	This work

Table S4. Literature examples of photocatalytic H_2 generation with hydrogenases under aerobic conditions.

H_2ase used	Catalytic System	O_2 Tolerance (based on)	Total H_2	Ref
[NiFeSe] *D. baculatum*	RuP-TiO_2, TEOA, pH 7.0, 25 °C, closed reactor	2.2% (1 h) (total H_2)	80 nmol TON 4,000 (1 h)	[7]
[NiFeSe] *D. baculatum*	Eosin Y, TEOA, pH 7.0, closed reactor	11±3% (TOF_{max})	66±27 nmol TON 6,600±2,700 (4 h)	[8]
Modified [NiFe] *E. coli*	g-C_3N_4/TiO_2, Ag nanocluster, TEOA, pH 6, closed reactor	17.5% (TOF_{max})	9.9 µmol TON 40,000 (2 h)	[12]
[NiFe] *T. roseopersicina*	$[Ru(bpy)_2(NH_2phen)](PF_6)_2$, MV EDTA, pH 5.5, closed reactor	11% (1 h) (total H_2)	19 nmol TON 50,000±300 (1 h)	[13]
[NiFe] *D. vulgaris*	$Ru(bpy)_3^{2+}$, porous glass plates (0.5 mm thick), MV, EDTA, pH 7.4, closed reactor	~71%, (TOF_{max})	34±13 µmol TON 130±60×10^3 (12 h)	[14]
[NiFeSe] *D. baculatum*	TiO_2, TEOA, pH 7.0, 80% vol. aq. glyceline, continuous air purge	88.5±12.7% (24.1 h) (total H_2)	21.3±3.0 µmol TON 1,020±144×10^3 (24.1 h)	This work
[NiFeSe] *D. baculatum*	TiO_2, TEOA, pH 7.0, 60% vol. aq. glyceline, continuous air purge	64.3±14.8% (24.1 h) (total H_2)	38.9±4.0 µmol TON 1,850±189×10^3 (72 h)	This work
[NiFeSe] *D. baculatum*	TiO_2, TEOA, pH 7.0, water continuous air purge	3.7±1.7% (24.1 h) (total H_2)	5.9±3.1 µmol TON 281±146×10^3 (72 h)	This work

Table S5. Solubility and diffusion coefficient of O_2 in different glyceline/water mixtures and observed O_2 tolerance of the photocatalytic H_2 evolution in these solvents after ~24 h.

Glyceline % Vol.	$c(O_2)$ mM	$D(O_2)$ 10^{-12} m^2 s^{-1}	$c(O_2) \times D(O_2)$ 10^{-12} mM m^2 s^{-1}	O_2 tolerance %
100	0.365±0.022	3.71±0.44	1.35±0.24	Not determined
80	0.303±0.056	17.5±3.2	5.29±0.97	88.5±12.7
60	0.295±0.054	40.3±7.3	11.7±2.2	64.3±14.8
40	0.292±0.054	84.8±15.1	23.9±4.4	20.5±3.2
30	0.291±0.053	128±22	35.4±6.5	16.0±6.8
20	0.290±0.053	211±36	56.2±10.3	6.5±1.5
10	0.289±0.053	431±76	106±19	3.5±3.3
0	0.289±0.001	2940±2	383±1	3.6±1.7

Table S6. Conditions and H_2 evolution performance by [NiFeSe]-TiO_2 in solutions of varying glyceline concentrations. Values observed after 24.1 h. ***** denotes total H_2 values after 24.9 h. Conditions: TiO_2 (5.0 mg), TEOA (0.4 M), *Db*[NiFeSe] H_2ase (21 pmol), 2.0 mL solvent, 40 °C, AM 1.5G, constant N_2 purge.

Glyceline % Vol.	H_2O – TEOA vol. mL	Total H_2 µmol	TON mol_{H2} mol^{-1}_{H2ase}	TOF_{max} mol_{H2} mol^{-1}_{H2ase} s^{-1}
80	0.4	24.9±0.6*	1.1±0.1 × 10^6*	14.1±2.2 after 5.2 h
60	0.8	59.5±5.3*	2.8±0.2 × 10^6*	54.2±1.7 after 1.6 h
40	1.2	68.1±3.0	3.2±0.1 × 10^6	56.8±6.8 after 2.4 h
30	1.4	72.4±2.0	3.4±0.1 × 10^6	78.3±3.0 after 0.8 h
20	1.6	91.4±12.0	4.4±0.5 × 10^6	122.4±10.1 after 0.8 h
10	1.8	85.7±10.5	4.1±0.5 × 10^6	144.7±45.8 after 0.8 h

Supporting References

[1] a) H. Shekaari, M. T. Zafarani-Moattar, A. Shayanfar, M. Mokhtarpour, *J. Mol. Liq.* **2018**, *249*, 1222-1235; b) S. Rozas, C. Benito, R. Alcalde, M. Atilhan, S. Aparicio, *J. Mol. Liq.* **2021**, *344*, 117717.

[2] A. Volbeda, P. Amara, M. Iannello, A. L. De Lacey, C. Cavazza, J. C. Fontecilla-Camps, *Chem. Commun.* **2013**, *49*, 7061-7063.

[3] a) M. G. Allan, M. J. McKee, F. Marken, M. F. Kuehnel, *Energy Environ. Sci.* **2021**, *14*, 5523-5529; b) J. Weber, A. J. Wain, F. Marken, *Electroanalysis* **2015**, *27*, 1829-1835.

[4] I. R. Krichevsky, *Zh. Fiz. Khim.* **1937**, *9*, 41−47.

[5] C. R. Wilke, P. Chang, *AIChE J.* **1955**, *1*, 264-270.

[6] E. Reisner, J. C. Fontecilla-Camps, F. A. Armstrong, *Chem. Commun.* **2009**, 550-552.

[7] E. Reisner, D. J. Powell, C. Cavazza, J. C. Fontecilla-Camps, F. A. Armstrong, *J. Am. Chem. Soc.* **2009**, *131*, 18457-18466.

[8] T. Sakai, D. Mersch, E. Reisner, *Angew. Chem. Int. Ed.* **2013**, *52*, 12313-12316.

[9] C. A. Caputo, L. Wang, R. Beranek, E. Reisner, *Chem. Sci.* **2015**, *6*, 5690-5694.

[10] G. A. M. Hutton, B. Reuillard, B. C. M. Martindale, C. A. Caputo, C. W. J. Lockwood, J. N. Butt, E. Reisner, *J. Am. Chem. Soc.* **2016**, *138*, 16722-16730.

[11] C. Tapia, S. Zacarias, I. A. C. Pereira, J. C. Conesa, M. Pita, A. L. De Lacey, *ACS Catal.* **2016**, *6*, 5691-5698.

[12] L. Zhang, G. Morello, S. B. Carr, F. A. Armstrong, *J. Am. Chem. Soc.* **2020**, *142*, 12699-12707.

[13] O. A. Zadvornyy, J. E. Lucon, R. Gerlach, N. A. Zorin, T. Douglas, T. E. Elgren, J. W. Peters, *J. Inorg. Biochem.* **2012**, *106*, 151-155.

[14] T. Noji, M. Kondo, T. Jin, T. Yazawa, H. Osuka, Y. Higuchi, M. Nango, S. Itoh, T. Dewa, *J. Phys. Chem. Lett.* **2014**, *5*, 2402-2407.
